# Sleep quality and neural circuit function supporting emotion regulation

**DOI:** 10.1186/2045-5380-2-22

**Published:** 2012-12-07

**Authors:** Jared D Minkel, Kristin McNealy, Peter J Gianaros, Emily M Drabant, James J Gross, Stephen B Manuck, Ahmad R Hariri

**Affiliations:** 1 Laboratory of NeuroGenetics, Department of Psychology & Neuroscience, Duke University, 417 Chapel Dr., Durham, NC 27708, USA; 2 Department of Psychology, University of Pittsburgh, Pittsburgh, Pennsylvania, USA; 3 Department of Psychology, Stanford University, Stanford, CA, USA; 4 Institute for Genome Sciences & Policy, Duke University, Durham, NC, 27708, USA

**Keywords:** Emotion, Emotion regulation, Sleep, Sleep quality, FMRI

## Abstract

**Background:**

Recent laboratory studies employing an extended sleep deprivation model have mapped sleep-related changes in behavior onto functional alterations in specific brain regions supporting emotion, suggesting possible biological mechanisms for an association between sleep difficulties and deficits in emotion regulation. However, it is not yet known if similar behavioral and neural changes are associated with the more modest variability in sleep observed in daily life.

**Methods:**

We examined relationships between sleep and neural circuitry of emotion using the Pittsburgh Sleep Quality Index and fMRI data from a widely used emotion regulation task focusing on cognitive reappraisal of negative emotional stimuli in an unselected sample of 97 adult volunteers (48 women; mean age 42.78±7.37 years, range 30–54 years old).

**Results:**

Emotion regulation was associated with greater activation in clusters located in the dorsomedial prefrontal cortex (dmPFC), left dorsolateral prefrontal cortex (dlPFC), and inferior parietal cortex. Only one subscale from the Pittsburgh Sleep Quality Index, use of sleep medications, was related to BOLD responses in the dmPFC and dlPFC during cognitive reappraisal. Use of sleep medications predicted lesser BOLD responses during reappraisal, but other aspects of sleep, including sleep duration and subjective sleep quality, were not related to neural activation in this paradigm.

**Conclusions:**

The relatively modest variability in sleep that is common in the general community is unlikely to cause significant disruption in neural circuits supporting reactivity or regulation by cognitive reappraisal of negative emotion. Use of sleep medication however, may influence emotion regulation circuitry, but additional studies are necessary to determine if such use plays a causal role in altering emotional responses.

## Background

Sleep problems are highly comorbid with psychiatric disorders [1,2] and have been found to predict the onset of both depressive episodes [3] and mania [4]. Although available data suggest a causal link between sleep and psychiatric symptoms, a compelling biological mechanism has not yet been reported. One hypothesis is that poor sleep compromises neural systems supporting emotion regulation [5]. Although this hypothesis was first based on clinical experience and anecdote, it has more recently been supported by independent lines of research that have established key neural and behavioral correlates of sleep deprivation in controlled laboratory experiments [6,7].

In general, these experiments have identified the prefrontal cortex, which plays a key role in the cognitive control of emotion [8], as particularly sensitive to sleep loss [9]. Experimental sleep deprivation studies have reported subjective [10], physiological [11], and neural [7] changes consistent with exaggerated responsiveness to negative emotional stimuli. The strongest support for this position comes from a study reporting decreased functional connectivity between the medial prefrontal cortex (mPFC) and amygdala in sleep deprived subjects viewing intense negative (i.e., disgusting and disturbing) photographs [7]. Although this emotion elicitation paradigm did not directly assess emotion regulation, the pathway involved shares considerable overlap with canonical emotion regulation circuitry [12], indicating that sleep deprivation results in deficient capacity to regulate strong emotional arousal. However, a serious limitation of studies to date is that they have used experimental paradigms with relatively long periods of sustained wakefulness that are very rare in real world settings.

In the present study, we extend the literature on the influence of sleep on neural substrates of emotion in three important ways. First, a well-characterized emotion regulation paradigm was administered during BOLD fMRI. This paradigm allows for the robust engagement of neural circuits, including the amygdala and mPFC, involved in emotional reactivity as well as cognitive reappraisal [13]. Cognitive reappraisal may be a particularly important process because it is one of the primary ways people can voluntarily alter their own emotional experiences [14] and is believed to represent a psychological mechanism by which people with psychiatric disorders improve mood and prevent relapse during cognitive behavior therapy [15,16]. To our knowledge, this is the first study to investigate relationships between sleep and emotion regulation per se. Second, self-reported sleep variables were collected using a widely utilized measure [17], allowing for the mapping of relationships between neural function associated with emotional regulation and individual differences in sleep variables commonly encountered in real-world settings. Third, our relatively large (N = 97), community-based sample allows greater generalizability than previous studies that have used smaller samples (less than 30), extreme levels of sleep deprivation rarely encountered in real-world settings, and young adult samples. Based on the existing literature, we hypothesized that sleep problems, particularly short sleep-duration, would predict deficits in neural activation supporting emotion regulation. Specifically, we predicted sleep problems would be associated with greater amygdala activation in response to negative emotional stimuli and lower activation in the mPFC during regulation of negative emotional stimuli.

## Methods

### Participants

Participants were recruited from Phase II of the Adult Health and Behavior project (AHAB II), which assesses a wide range of behavioral and biological traits among middle-aged community volunteers. All participants had completed both the emotion regulation fMRI task and the Pittsburgh Sleep Quality Index (PSQI) and were in good general health. The University of Pittsburgh Institutional Review Board approved the study and all participants provided informed consent in accordance with its regulations. Participants were evaluated for current *DSM-IV* Axis I disorders using the Mini-International Neuropsychiatric Interview (MINI [18]) and excluded only for history of psychosis. The participants were free of medical diagnoses of cancer, stroke, diabetes requiring insulin treatment, and chronic kidney or liver disease. Additional exclusion criteria included use of psychotropic, glucocorticoid, hypolipidemic, antiarrhythmic, antihypertensive, and prescription weight loss medication. Sleep medications were allowed if they were not taken more than 7 of 14 days prior to eligibility determination.

Our initial sample included 106 unselected participants, but 8 were removed for amygdala coverage less than 90%. One additional participant was removed for abnormally high motion artifact, leaving a final sample of 97 participants (48 women; mean age 42.78 ± 7.37 years, range 30–54 years old).

### Measures

#### Pittsburgh Sleep Quality Index (PSQI)

All participants completed the PSQI, a 19-item self-rated questionnaire for evaluating general sleep patterns over the previous month [17]. The questionnaire is scored to produce seven clinically-derived component scores, each of which is converted to a 0–3 scale where higher numbers indicate more problematic sleep. Many of the component scores are based on a single item or reflect a single calculation based on two or more items. The component scores are subjective sleep quality, sleep latency, sleep duration, habitual sleep efficiency (a measure of time spent asleep to total time spent in bed), sleep disturbances, use of sleeping medication, and daytime dysfunction. The combined score is reported as Global Sleep Quality. Global scores greater than 5 indicate clinically meaningful sleep disturbance.

#### Center for Epidemiologic Studies Depression Scale (CES-D)

To ensure that observed relationships were not better accounted for by co-occurring symptoms of mood disorders, depression symptoms were also evaluated using the CES-D [19]. The CES-D is a 20-item measure of depression symptoms that has been evaluated and used in many studies of psychiatric symptoms and disorders. A cutoff score of 16 has been used to differentiate those who are likely to have clinically meaningful levels of depressive symptoms from those who are not [20].

#### Emotion regulation paradigm

The emotion regulation task in this experiment was adapted from a previously validated paradigm [8,13]. The task consisted of 30 negative photographs and 15 neutral photographs selected from the International Affective Picture System (IAPS) database based on published norms [21]. Negative photographs depicted bodily illness and injury (21 photographs), acts of aggression (3 photographs), members of hate groups (2 photographs), transportation accidents (2 photographs) and human waste (2 photographs). Neutral photographs depicted inanimate objects (10 photographs) or neutral scenes (5 photographs).

Prior to completing the task, subjects were instructed that when cued to “look,” they were to maintain attention on the stimulus and allow their emotional reaction to occur without attempting to change it. When cued to “decrease,” they were to attempt to reduce their emotional response through cognitive reappraisal (i.e., by thinking of something that makes the photograph seem less negative). Subjects were given examples of reappraisal strategies for specific photographs and then practiced the skill outside the MRI scanner. During the MRI scan each trial consisted of a 2 second cue to either “look” or “decrease” one’s emotional response, then a 7 second presentation of either a negative or neutral picture, then a 4 second opportunity to rate the picture, followed by a 1–3 second rest period before the next cue. During the “rate picture” phase, subjects were instructed to report their emotional reaction to each photograph on a scale of 1 to 5, where 1 indicated neutral and 5 indicated feeling strongly negative. The ratings were made using a button response pad in the participant’s right hand and recorded in E-Prime software.

Fifteen negative photographs were presented with the “look” cue and 15 were presented with the “decrease” cue. All 15 neutral photographs were presented with the “look” cue (because there is nothing to regulate in response to a neutral photograph). The stimuli were presented in pseudo-random order such that no more than 2 of the same instruction (look vs. regulate) could be presented consecutively and no more than 4 negative stimuli could be presented consecutively. Total time for the task was 11:28 minutes. This design allows for an assessment of neural activation related to the emotional valence of the stimuli (look negative > look neutral) as well as activation related to reappraisal (regulate negative > look negative).

### BOLD fMRI data acquisition

Each participant was scanned using a Siemens 3 T Allegra scanner (Siemens AG, Medical Solutions, Erlangen, Germany) developed specifically for advanced brain imaging applications and characterized by increased T2* sensitivity and fast gradients that minimize echo spacing, thereby reducing echo-planar imaging (EPI) geometric distortions and improving image quality. An autoshimming procedure was conducted to minimize field inhomogeneities. A series of 34 interleaved axial slices aligned with the AC-PC plane were acquired with a gradient-echo echo planar imaging sequence (TR/TE = 2000 ms/25 ms; FOV = 200 mm, matrix size 64 × 64; 3.125 × 3.125 × 3 mm voxels; interslice skip = 0). Two initial RF excitations were performed (and discarded) to achieve steady-state equilibrium. However, the first two acquired volumes were discarded during preprocessing to further ensure steady-state equilibrium. All scanning parameters were selected to optimize the quality of the BOLD signal while maintaining a sufficient number of slices to acquire whole brain data. Before the collection of fMRI data for each participant, we acquired a reference EPI scan that we visually inspected for artifacts (e.g., ghosting) as well as good signal across the entire volume of acquisition.

### BOLD fMRI data analysis

Whole-brain image analysis of all fMRI data was conducted at the Laboratory of NeuroGenetics at Duke University using the general linear model (GLM) of SPM8 ( http://www.fil.ion.ucl.ac.uk/spm). Images for each participant were realigned to the first volume in the time series to correct for head motion, spatially normalized into a standard stereotactic space (Montreal Neurological Institute template) using a 12-parameter affine model (final resolution of functional images = 2 mm isotropic voxels), and smoothed to minimize noise and residual difference in gyral anatomy with a Gaussian filter, set at 6-mm full-width at half-maximum. Preprocessed data sets were analyzed using second-level random-effects models that account for both scan-to-scan and participant-to-participant variability to determine task-specific regional responses.

Variability in single-subject whole-brain functional volumes was determined using the Artifact Recognition Toolbox ( http://www.nitrc.org/projects/artifact_detect). Individual whole-brain BOLD fMRI volumes meeting at least one of the following two criteria were identified as artifacts during the determination of task-specific effects: 1) significant mean-volume signal intensity variation 2(i.e., within volume mean signal greater or less than 4 standard deviations of mean signal of all volumes in time series), and 2) individual volumes where scan-to-scan movement exceeded 2 mm translation or 2° rotation in any direction. Artifacts were then treated as regressors of no interest in subsequent preprocessing steps. One participant was removed from further analyses due to abnormally high artifact (37% of whole-brain BOLD fMRI volumes). The remaining participants had, on average, 2.64% of all volumes identified as artifacts, thus we believe this approach enhanced our capacity to determine task-specific effects by minimizing the influence of volumes with substantial variability without compromising our power to detect task-specific effects by excluding a large number of volumes.

Following preprocessing, linear contrasts employing canonical hemodynamic response functions were used to estimate condition-specific (i.e. negative > neutral and regulate > look) BOLD responses for each individual. Individual contrast images (i.e., weighted sum of the beta images) were then used in second-level random effects models accounting for scan-to-scan and participant-to-participant variability to determine mean condition-specific regional responses using one-sample t-tests. A voxel-level statistical threshold of *p* < 0.05, FWE corrected for multiple comparisons (across the anatomical amygdala ROIs for negative > neutral, and across whole brain for regulate > look) was applied. An additional extent threshold of 10 contiguous voxels was applied to both ROI and whole brain analyses.

Based on previous findings [7,13,22] we selected the amygdala as a region of interest (ROI) where we expected to find effects of emotional reactivity (negative > neutral contrast). A bilateral amygdala ROI mask was created from the Automated Anatomical Labeling (AAL) atlas [23]. Because of the potential for signal loss and noise often observed in the amygdala and adjacent regions, single-subject BOLD fMRI data were included in subsequent analyses only if there was a minimum of 90% signal coverage in the amygdala masks bilaterally. Although we anticipated finding main effects of task in regions previously identified [8,13], these analyses were completed using whole brain analyses. No additional ROI masks were created.

BOLD parameter estimates from clusters exhibiting condition-effects (negative > neutral; regulate > look) were extracted using the VOI tool in SPM8 and exported for analyses in R and SPSS (v.18). Extracting parameter estimates from functional clusters activated by our fMRI paradigm, rather than clusters specifically correlated with our independent variables of interest, precludes the possibility of any correlation coefficient inflation that may result when an explanatory covariate is used to select a region of interest [24]. We have used this more conservative and rigorous analytic strategy in recent studies [25,26].

### Statistical analyses

The influence of sleep duration on emotional reactivity and emotion regulation was investigated using regression analyses. Because sleep duration was measured prior to the emotion regulation task and because it is believed to play a causal role in influencing subjective responses and neural responses during emotional reactivity and regulation, it was treated as an independent variable in all regression analyses. Dependent variables included subjective emotional responses to photographs as well as extracted BOLD parameter estimates from max voxels of clusters showing significant condition-effects and ratings of emotional reactions during each condition. Dependent variables were analyzed to ensure they were approximately normally distributed prior to entry into regression analyses. Relationships among subjective emotional reactions and extracted BOLD parameter estimates were investigated using Pearson correlations. Because we made a priori predictions that poorer sleep would predict less brain activation during emotion regulation and less subjective regulatory success, one-tailed significance tests were used in these analyses. For all other analyses, two-tailed significance tests were used.

## Results

### Negative emotion induction and regulation manipulation checks

#### Emotional reactivity

Subjective responses to photographs were compared using paired samples t-tests. Two subjects were excluded based on abnormal or missing self-report data. These analyses confirmed that negative photographs elicited significantly greater negative emotion than neutral photographs (*t*(95) = 37.02, *p* < .001).

**Figure 1:**
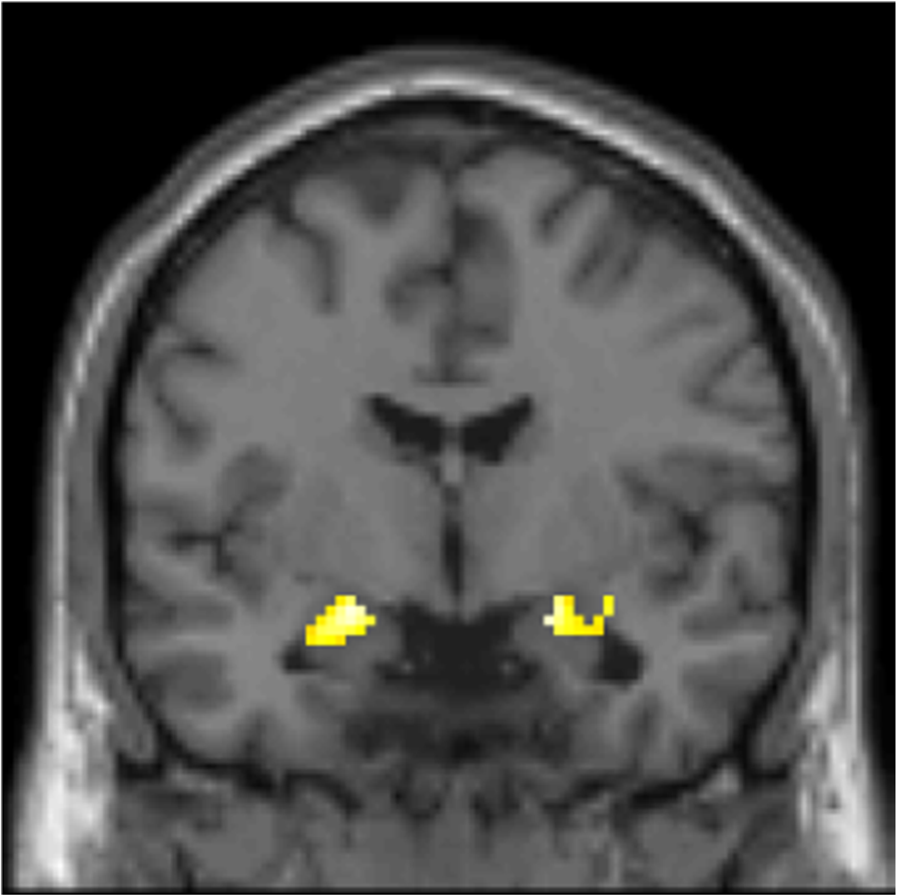
**Statistical parametric map illustrating amygdala activation associated with viewing IAPS photographs (look negative > look neutral).** Maximal voxel MNI coordinates for left amygdala activation: -20, -6, -14; 89 voxels, *t*=5.73, *p*<.001. Maximal voxel MNI coordinates for right amygdala activation: 20, -4, 16; 39 voxels, *t*=5.00, *p*<.001.

Based on previous findings [7,22], we focused our analyses of neural responses associated with emotional reactivity (negative > neutral contrast) on amygdala activation. A significant condition-effect was found in both the left (89 voxels, *t* =5.73, *p* < .001; -20, -6, -14) and right (39 voxels, *t* =4.70, *p* < .001; 20, -4, -16) amygdala (Figure 1).

#### Emotion regulation

Cognitive reappraisal was associated with significantly less intense subjective negative emotion than simply viewing negative photographs (*t*(95) = 9.65, *p* < .001). These results confirmed that the emotion regulation paradigm produced the intended subjective effects and that participants were able to regulate their emotional responses through reappraisal.

**Figure 2:**
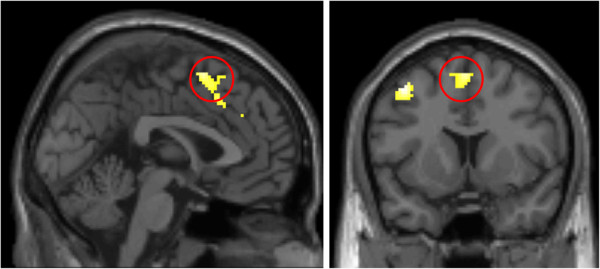
**Statistical parametric map illustrating whole brain activation associated with reappraisal of emotional responses to negative IAPS photographs (regulate negative > look negative).** The cluster in the dorsal medial prefrontal cortex (mPFC; within the red circle) showed a significant relationship with regulatory success based on subjective responses to IAPS photographs (*r*=.21, *p*=.04). Maximal voxel MNI coordinates for medial prefrontal cluster: 0, 10, 60; 241 voxels, *t*=6.32, *p*<.001. The cluster in the left dorsolateral prefrontal cortex (dlPFC), maximal voxel MNI coordinates for dlPFC cluster: -42, 8, 52; 198 voxels, *t*=7.11, *p*<.001, showed a marginally significant relationship with regulatory success (*r*=.18, *p*=.07). A third cluster (not visible here) was located in the inferior parietal cortex and had maximal voxel MNI coordinates -50, -58, 46; 481 voxels, *t*=6.98, *p*<.001.

Analyses of neural responses associated with cognitive reappraisal (regulate > look contrast) revealed significant main effects of task in three clusters located within the medial prefrontal cortex (mPFC), left dorsolateral prefrontal cortex (dlPFC), and inferior parietal cortex (IPC, Figure 2). Regulatory success (computed as the difference between subjective responses in the regulate negative and look negative conditions) was significantly correlated with activation in the mPFC (*r* = .206, *p* = .044) and IPC (*r* = .213, *p* = .037) and trended toward a significant relationship with activation in the left dlPFC cluster (*r* = .184, *p* = .073), indicating that greater activation in these clusters was associated with more effective use of reappraisal, as indexed by lower overall subjective emotional responses to the negative stimuli.

### Sleep quality analyses

#### Sleep and emotional reactivity

Sleep was measured using the Pittsburgh Sleep Quality Index (see Table 1 for frequency and severity of sleep problems). Global Sleep Quality, i.e., the composite score reflecting the sum of all subscales) was analyzed to determine overall relationships among sleep and emotional reactivity (see Table 1 for PSQI score descriptive statistics). Global Sleep Quality scores did not predict left (*b = −*.135, *p* = .19) or right (*b = −*.044, *p* = .67) amygdala activation during the negative > neutral contrast. Global PSQI scores were also unrelated to subjective responses to negative vs. neutral photographs (*b = −*.134, *p* = .193). Neither sleep duration nor sleep quality predicted left (*ps* > .59) or right (*ps* > .41) amygdala activation in the negative > neutral contrast. Similarly, there was no relationship between PSQI subscales and subjective emotional responses to negative vs. neutral photographs in the “look” condition (*ps* > .29), except for a trend toward poorer subjective sleep quality predicting *less* intense subjective emotional responses (*b = −*.189, *p* = .066).

**Table 1:** Pittsburgh Sleep Quality Index Component Scores

		**Frequency (N=97)**			
**Subscale**	**M (SD)**	**Normal 0**	**Mild 1**	**Moderate 2**	**Severe 3**
*Subjective Sleep Quality*	.94 (.67)	24	56	16	1
*Sleep Latency*	.85 (.73)	31	53	10	3
*Sleep Duration*	.87 (.64)	26	59	11	1
*Habitual Sleep Efficiency*	.30 (.64)	76	14	6	1
*Sleep Disturbances*	1.31 (.53)	1	67	27	2
*Use of Sleeping Medication*	.18 (.50)	85	7	5	0
*Daytime Dysfunction*	.71 (.56)	33	59	5	0

#### Sleep and emotion regulation

**Figure 3:**
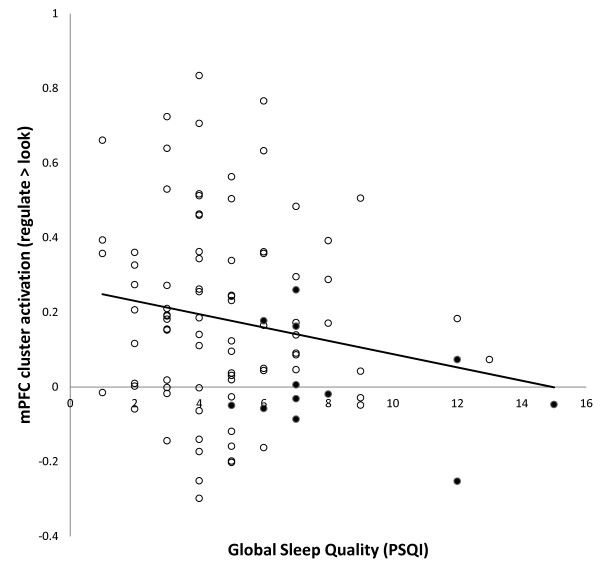
**Scatter plot showing individual differences in activation in the medial prefrontal cortex (mPFC) for the regulate negative > look negative contrast based on global sleep quality scores for the PSQI.** Worse sleep predicted less mPFC cluster activation (b = -.190, *p* = .03). Filled circles indicate participants who reported using sleep medication within the last month.

**Figure 4:**
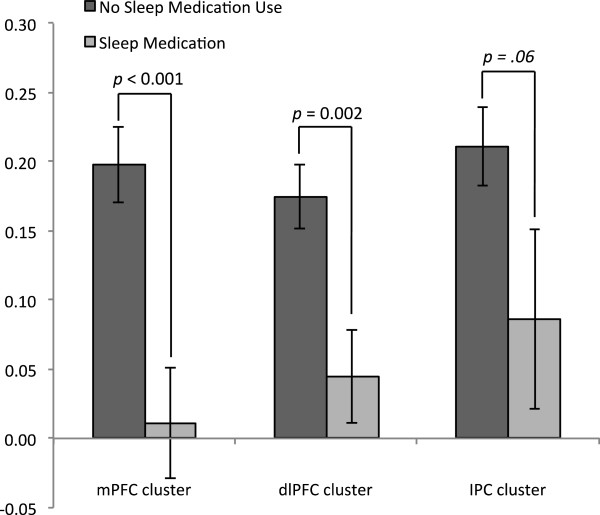
**Activation during the regulate negative > look negative condition was greater for those who denied use of sleep medication in the last 30 days (dark gray bars, n = 85) than for those who endorsed it (light gray bars, n = 12).** Differences were significant in the medial prefrontal cluster (mPFC, *p* = .001) and left dorsolateral prefrontal cluster (dlPFC, *p* = .004) and there was a non-significant trend in the inferior parietal cortex cluster (IPC, *p* = .06).

Global PSQI scores predicted activation in the mPFC cluster, (*b = −*.190, *p* = .03) such that worse sleep was associated with less mPFC activity (Figure 3). Global PSQI scores were not significantly related to activation in the dlPFC cluster (*b = −*.052, *p* = .31) or the IPC cluster (*r* = .003, *p* = .49). Similarly, Global PSQI scores were not significantly related to self-reported regulatory success (*r* = .008, *p* = .47). Analyses of PSQI subscales revealed a significant relationship between use of sleep medication and activation in the mPFC cluster (*b = −*.23, *p* = .02), left dlPFC cluster (*b = −*.22, *p* = .02) and IPC cluster (*b = −*.196, *p* = .03). Use of sleep medication did not predict self-reported regulatory success however (*b =* 0.017, *p* = .44). Use of sleep medication was relatively rare with 12 subjects (6 women) reporting such use in the last month. Of these, 7 reported using sleep medication less than once per week and 5 reported using sleep medications 1–2 times per week. Due to these small groups, they were combined and compared to those who did not use sleep medications (n = 85, 42 women). Independent samples t-tests confirmed that those who used sleep medication demonstrated less activation in the mPFC cluster (*t*(23.1) = 3.85, *p* < .001) and dlPFC cluster (*t*(23.2) = 3.17, p = .002) than those who did not use sleep medications, but differences in activation in the IPC cluster showed a non-significant trend (*t*(95) = 1.56, *p* = .06; Figure 4). All other PSQI subscales failed to predict activation in the dlPFC cluster (all *b* < .12, *ps* > .26), mPFC cluster (all *b* < .11, *ps* > .16), or IPC cluster (all *b* < .10, *ps* > .20). Furthermore, controlling for all other PSQI subscales had no effect on relationships between use of sleep medications and neural activation supporting emotion regulation (mPFC cluster: *b* = −.20, *p* = .04, dlPFC: *b* = −.24, *p* = .02, IPC cluster: *b* = −.24, *p* = .02). Removing participants who reported using sleep medication from the analyses resulted in a non-significant relationship between Global Sleep Quality and activation in the mPFC (*b* = −0.75, *p* = .50).

One final analysis we conducted was to create a composite score of all PSQI subscales except medication use. Here we included all participants, including those who reported using sleep medication, but did not allow this medication use to affect their Global PSQI score. The relationship between mPFC activation and this composite sleep score was nearly significant (b = 0.16, p = .06) suggesting that it may have not been the use of sleep medication per se that accounted for this relationship.

### Control analyses

Additional analyses were completed in order to ensure that our findings related to sleep and emotion were not better accounted for by mood problems. Depression symptoms, measured by the CES-D, were highly correlated with PSQI Global scores (*r* = .56, *p* < .001), but were not significantly correlated with activation in the clusters identified above (*ps* > .20). Similarly, subjects scoring above the CES-D cutoff score of 16 (n = 14) did not show significantly different activation relative to those scoring below the cutoff score in any of the clusters identified above (*ps* > .62). There was a non-significant trend toward greater depression symptoms predicting less self-reported regulatory success (*b = −*.177, *p* = 0.09).

## Discussion

Inadequate sleep is thought to produce exaggerated emotional responses by compromising the top-down inhibitory control of the prefrontal cortex over amygdala driven emotional arousal [5-7]. If true, this could be a promising mechanism that contributes to relatively high rates of depression in industrialized and developing nations. Our findings suggest however, that findings from experimental settings, where wakefulness can be extended for long durations, were not replicated in our sample where more modest variability in sleep was observed. Because this was a large, unselected sample from the community, we believe the findings are more relevant for public policy than smaller self-selected samples typical of experimental studies. Although there was a significant relationship between the composite PSQI score Global Sleep Quality and neural activation in the mPFC, analyses of subscales showed that most aspects of sleep were unrelated to emotional functioning. Perhaps most important was the finding that there were no significant relationships between emotional responses to negative photographs and either how much participants slept (sleep duration) or their reports of how well they slept (subjective sleep quality). We interpret these findings as evidence that the human nervous system can preserve regulatory efficiency of negative emotion within the variability in sleep observed in the general population. Because our paradigm measured emotional reactivity with and without voluntary regulation strategies, we believe these findings are particularly robust. It is important to emphasize, however, that these findings cannot necessarily be extended to clinical populations. There is substantial evidence that relationships between sleep and emotion may be very different in people with psychiatric diagnoses such as Bipolar Disorder and Major Depression [4,27] and additional investigations of underlying biological mechanisms to explain those differences are needed. One final caveat is that in an unselected sample, people are free to choose their own sleep schedule and those who are most sensitive to sleep loss may choose to sleep more than those who are more resilient. The emotional consequences of sleep loss may be more profound in subgroups that cannot choose their own sleep schedules, such as emergency personnel and shift workers. Additional studies are needed to investigate possible emotional consequences of abnormal sleep in these populations.

Our secondary analyses suggested that the relationship between the composite PSQI score Global Sleep Quality and neural activation during cognitive reappraisal was primarily driven by the PSQI subscale related to sleep medications. Use of sleep medication was associated with reduced activation in both the dorsolateral prefrontal and medial prefrontal clusters. This finding, if replicated, would suggest that sleep medication should be used with caution, especially in people with a predisposition for mood problems. We would like to emphasize, however, that this was an unexpected finding from a correlational study that cannot establish a causal relationship between sleep medication use and the abnormalities identified. In addition, data were not available on the class of sleep medications these participants used, so additional studies are needed to investigate what influence, if any, different classes of sleep medication have on emotion regulation circuitry. It is also possible that a third variable can explain both sleep medication use and abnormal neural responses during the emotion regulation task. Perhaps the most likely such variable, namely depressive symptoms, was ruled out by our analyses. There was no relationship between depression and neural substrates of emotion regulation in this community sample. Future studies could extend these findings considerably by evaluating emotion regulation before and after the administration of sleep medications. Because the participants who used sleep medication also reported greater sleep disturbances overall, it is further possible that this relationship more generally reflects a negative impact of moderate to severe sleep disturbance on the engagement of prefrontal regions during cognitive reappraisal of emotional stimuli.

The analyses reported above have several important limitations we would like to emphasize. First, we estimated average sleep duration using self-report, where objective methods such as actigraphy or polysomnography would have been more accurate, suggesting that we may have failed to detect a true correlation. Our relatively large sample size partially attenuates this concern, but future studies might be able to detect small, but statistically significant effects through actigraphy or polysomnography. Second, our sample consisted of adults in the age range of 30 to 54 years. There is reason to believe that sleep may have different effects on emotion throughout the lifespan [5,10] and additional studies of different age groups are needed to better understand these relationships in children, adolescents, and the elderly. Finally, although depression symptoms were measured and could therefore be evaluated as possible confounds, there were many other potential confounds, such as stress or anxiety, that we could not evaluate in this study.

## Conclusions

We believe that the analyses presented above demonstrated two important findings. First, they suggest that healthy normal adults can cope with mild to moderate sleep problems without showing large changes in neural or subjective emotional functioning, at least in response to passive experiences of negative stimuli. Nevertheless, only a handful of studies have investigated the neurobiological bases of emotional changes associated with sleep and more research is needed to understand main effects and individual differences in affective responses to sleep loss. Second, we found evidence suggesting that taking sleep medications may result in altered neural function supporting emotion regulation, and that these alterations may more broadly reflect the negative impact of moderate to severe sleep disturbances. Although preliminary, we believe this finding justifies additional studies designed to evaluate the impact of sleep medications on emotion regulation.

## Competing interests

The authors report no biomedical financial interests or potential conflicts of interest.

## Authors’ contributions

JDM conceived of the analyses reported here, completed statistical analyses and drafted the manuscript. KM completed first level MRI analyses and consulted on additional analyses. PJG participated in the design of the emotion regulation task and helped to draft the manuscript. JJG provided expertise in designing the emotion regulation task and helped to draft the manuscript. SBM participated in the design of the experiment and coordinated data exchange between sites. ARH participated in the design of the experiment, co-wrote the manuscript, and provided mentorship for all analyses completed. All authors read and approved the final manuscript.
